# Distinct and redundant functions of cyclin E1 and cyclin E2 in development and cancer

**DOI:** 10.1186/1747-1028-5-2

**Published:** 2010-01-17

**Authors:** C Elizabeth Caldon, Elizabeth A Musgrove

**Affiliations:** 1 Cancer Research Program, Garvan Institute of Medical Research, Sydney, NSW 2010, Australia; 2 St Vincent's Clinical School, Faculty of Medicine, University of NSW, NSW 2052, Australia

## Abstract

The highly conserved E-type cyclins are core components of the cell cycle machinery, facilitating the transition into S phase through activation of the cyclin dependent kinases, and assembly of pre-replication complexes on DNA. Cyclin E1 and cyclin E2 are assumed to be functionally redundant, as cyclin E1^-/- ^E2^-/- ^mice are embryonic lethal while cyclin E1^-/- ^and E2^-/- ^single knockout mice have primarily normal phenotypes. However more detailed studies of the functions and regulation of the E-cyclins have unveiled potential additional roles for these proteins, such as in endoreplication and meiosis, which are more closely associated with either cyclin E1 or cyclin E2. Moreover, expression of each E-cyclin can be independently regulated by distinct transcription factors and microRNAs, allowing for context-specific expression. Furthermore, cyclins E1 and E2 are frequently expressed independently of one another in human cancer, with unique associations to signatures of poor prognosis. These data imply an absence of co-regulation of cyclins E1 and E2 during tumorigenesis and possibly different contributions to cancer progression. This is supported by *in vitro *data identifying divergent regulation of the two genes, as well as potentially different roles *in vivo*.

## Introduction

**Figure 1:**
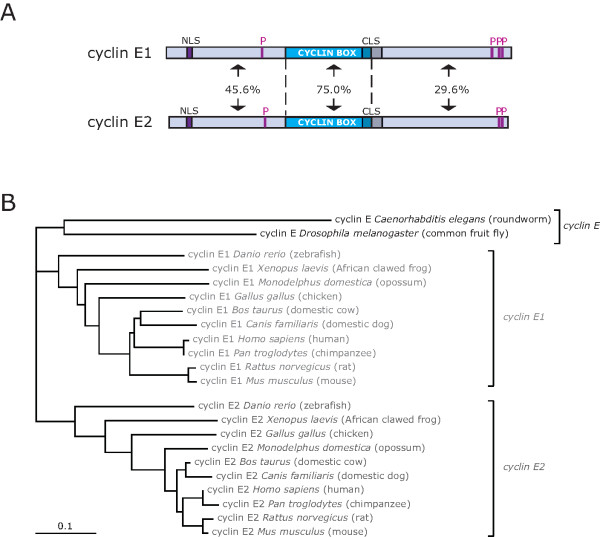
**Cyclin E1 and cyclin E2 are similar proteins, but are independently conserved in vertebrate organisms**. **A**. *Homo sapiens *cyclin E1 and cyclin E2 proteins were aligned and percentage similarity calculated using ALIGN [127]. The sequences have 48.6% identity overall, with higher identity within the well-conserved cyclin box (75.0%), and less conservation in the N-terminal (45.6%) and C-terminal regions (29.6%). NLS = nuclear localisation sequence, CLS = centrosome localisation sequence, P = phosphorylation site. **B**. Cyclin E from invertebrates was compared to cyclin E1 and cyclin E2 from several vertebrate organisms. The sequences were aligned using CLUSTALW and the GONNET matrix [128], and a phenogram derived of the alignment using the DRAWTREE application of the PHYLIP package [129]. The phenogram was visualised with the application TREEVIEW [130]. The scale bar indicates 0.1 amino acid changes per character.

Cyclin E1, the prototypic E-cyclin, was first described in 1991 [1], and has since been found to have crucial roles in cell proliferation and oncogenesis [2,3]. The second mammalian E-cyclin, cyclin E2, was identified in 1998 [4,5], and is largely regarded as being functionally redundant with cyclin E1 [2,3,6]. Cyclin E1 and cyclin E2 are encoded by different genes: cyclin E1 by CCNE1 at 19q12, and cyclin E2 by CCNE2 at 8q22.1. The cyclin E1 and cyclin E2 proteins display high sequence similarity (69.3% in *Homo sapiens*), and important functional motifs are conserved. These include domains for Cdk (cyclin dependent kinase) and Cdk inhibitor interaction, a nuclear localisation sequence and a centrosome localisation sequence (Figure 1). This high sequence conservation has supported a hypothesis of complete redundancy between the two proteins.

More recent data have identified instances of specific regulation or function for each E-cyclin. First, animal models hint that we have not fully delineated the roles of the E-cyclins, and cyclin E2^-/- ^mice display subtle phenotypes that may indicate key functional differences to cyclin E1. A second difference is that cyclins E1 and E2 can be regulated by distinct transcription factors and miRNAs. Finally, the expression of cyclin E1 and E2 is not always linked in cancer, and this discordance confirms that there are likely to be underlying functional and regulatory differences between the two proteins.

## Known functions of the E-cyclins

The E-type cyclins activate the kinase Cdk2 that phosphorylates substrates including the retinoblastoma protein (Rb). Rb phosphorylation leads to the release of E2F transcription factors and initiation of S phase and DNA synthesis, by induction of expression of S phase proteins including histone proteins and cyclin A. Cyclin E-Cdk2 also directly phosphorylates proteins involved in centrosome duplication (NPM, CP110, Mps1), DNA synthesis (Cdt1), DNA repair (Brca1, Ku70), histone gene transcription (p220/NPAT, CBP/p300, HIRA) and Cdk inhibitors p21^Waf1/Cip1 ^or p27^Kip1 ^(reviewed in [2,3,7]). The specificity of cyclin-Cdk activity towards particular substrates is predominantly mediated via differences in cyclin sequence and periodic expression of cyclins during cell cycle phases, along with specific sub-cellular localisation [8,9]. Given that cyclins E1 and E2 are very similar in sequence and are both nuclear proteins [5,10], it seems probable that cyclin E1-Cdk2 and cyclin E2-Cdk2 phosphorylate a very similar subset of proteins so long as they exhibit the same periodicity of expression. There is considerable overlap even between cyclin E1-Cdk2 and cyclin A-Cdk2 targets [8]. Cyclin E1 can also activate Cdc2/Cdk1. In Cdc2 knockout mice, cyclin E1-Cdc2 kinase activity compensates for the absence of cyclin E1-Cdc2 activity to promote S phase entry [11]. Cyclin E1 also interacts with Cdc2 in the presence of Cdk2, which possibly contributes to S-phase entry in mitotic cell cycles [11]. Both E-cyclins can also complex with Cdk3, although it is not known if this interaction is significant *in vivo *[5,12].

**Figure 2:**
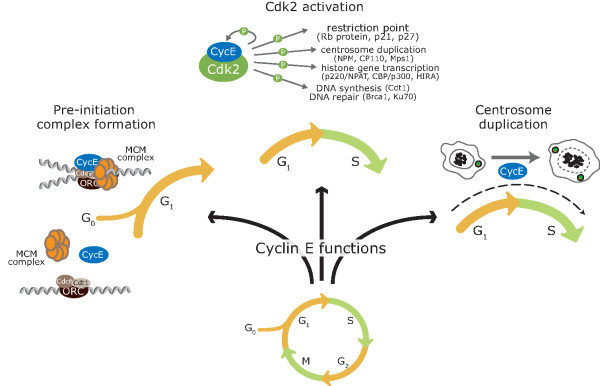
**Cyclin E has multiple functions in cell cycle progression, both Cdk-dependent and Cdk-independent**. Cyclin E is necessary for the formation of pre-replication complexes on DNA as cells re-enter the cell cycle after quiescence. Cyclin E also activates the Cdk2 holoenzyme, and phosphorylates many targets at the G_1 _to S phase transition of the cell cycle, including the retinoblastoma protein (Rb). Finally, cyclin E, via its CLS binding motif, interacts with centrosomes and promotes centrosome duplication.

While a predominant function of the E-cyclins is to activate Cdk2, it has become apparent that there are crucial Cdk-independent roles (Figure 2). Cdk2^-/- ^mice are viable whereas cyclin E1^-/- ^E2^-/- ^mice are embryonic lethal [13], implying an essential Cdk-independent function for the E-cyclins. Furthermore, truncated variants of cyclin E1 that cannot bind Cdk2 are able to induce malignant transformation [12], and oncogenesis has been associated with increased cyclin E1 in the absence of increased Cdk2 activity [12,14-16]. Subsequent to these studies, additional major roles for cyclin E1 have been established in the formation of pre-replication complexes on DNA, endocycling and centrosome duplication (Figure 2).

In order for DNA synthesis to occur after the transition from quiescence (G_0_) into the cell cycle, it is necessary for the DNA replication complexes to be assembled *de novo *at the origins of replication. These pre-initiation replication complexes (pre-RC) consist of the pre-licensing factors Cdt1 and Cdc6 that associate with the origin-binding protein ORC, and the DNA replicative helicase components, MCM 2-7 [17]. Cyclin E1, independent of Cdk2 activity, associates with DNA near replication origins, and facilitates MCM loading at origins through direct interactions with MCM proteins and Cdt1 [18]. This process may also be important in endoreplication, where DNA is replicated without cell division. Endoreplication results in polyploid cells that have essential functions in development, cell differentiation and as an energy reserve [19]. E-cyclins are crucial for endocycling, with the absence of E-cyclins leading to reduced DNA copy number and mortality due to failures in the polyploid giant trophoblast cells of the placenta [13,20]. Like diploid cells, endocycling cells require pre-RC assembly at origins of replication. While the precise function of the E-cyclins in endocycling is not established, in *Drosophila *cyclin E recruits MCM2 to the DNA during early endocycles of polytene cells of the salivary gland [21], implying that E-cyclins also function in pre-RC formation in these cells.

A 20 amino acid centrosome localisation sequence (CLS) in cyclin E1 targets this protein to the centrosome [22]. Cyclin E1 overexpression increases the proportion of cells in S phase by a mechanism that is dependent upon this region, but independent of Cdk2-binding [22]. The increase in the proportion of S phase cells may reflect a lengthening of S phase, rather than an increase in proliferation *per se *[23]. In fact, the cyclin E1 CLS is responsible for co-localising MCM5 to the centrosome, where its presence inhibits centrosome over-duplication and proliferation [24]. Consequently the CLS may function as a coordinating link between centrosome duplication and pre-RC formation [24]. Cdk2 activity is also synchronised with these events, as cyclin E1-Cdk2 phosphorylates nucleophosmin, CP110 and Mps1, promoting centrosome duplication [2,3,7].

The non-Cdk functions of the E-cyclins have been investigated using cyclin E1, and have been presumed to be identical for cyclin E2 [18,22]. The CLS motif is well conserved between cyclin E1 and cyclin E2, implying that cyclin E2 would be functionally active at the centrosome [18,22]. Cyclin E1^-/- ^E2^-/- ^mice are embryonic lethal whereas the individual knockout mice have largely normal phenotypes, which supports an assumption that either cyclin E1 or E2 can fulfil all of the functions of the E-cyclins. Despite this apparent redundancy, a careful consideration of animal models leads us to question whether cyclin E1 and cyclin E2 are true homologs.

## E-cyclins in animal models

### Non-canonical functions of "Cyclin E" in developmental models

Both E-cyclins are expressed in vertebrates, whereas invertebrates such as *Drosophila melanogaster *and *Caenorhabditis elegans *each have only one "cyclin E", which is essential for viability [25,26]. Cyclin E1, but not cyclin E2, is maternally expressed in *Xenopus laevis *embryogenesis, such that cyclin E1 is the only E-type cyclin during early embryonic cell cycles. The presence of a single E-cyclin has allowed for sophisticated studies of E-cyclins in these organisms that are not confounded by functional compensation between the E-cyclins. Consequently some of the major roles of E-cyclins, including activation of Cdk2 during the G_1 _to S phase transition [26], endoreplication [27], and formation of pre-RCs [21,28], were identified very early in these models.

Studies in these organisms have also hinted that E-type cyclins may have roles in addition to those described in pre-RC formation, Cdk activation and centrosome biology. In embryonic cell cycles that lack G_1 _and G_2 _phases, E-cyclins are expressed throughout the cell cycle and particularly during S phase [29], and this is associated with high Cdk2 activity [30], and normal progression through S phase. By contrast, in the somatic cell cycles of mammalian cells, the expression of the E-cyclins is confined to a window between late G_1 _and early S phase [4,5,10], and the degradation of cyclin E1 during S phase is a pre-requisite for mitosis and entry into the next cell cycle [23,31]. Cyclin E1-Cdk2 activity is particularly high on the mitotic chromosomes of cells in early *Xenopus *embryos, which suggests that cyclin E may be promoting pre-RC assembly directly after mitosis [32]. An alternative explanation is that cyclin E may facilitate DNA replication fork movement in certain circumstances. A *Drosophila *mutant has been identified in which a mutation in the cyclin E gene increases replication fork movement in polytene chromosomes [33]. Cyclin E1 accumulates on chromatin during S phase in *Xenopus *extracts [28], potentially with a role in Cdk2-mediated chromatin decondensation for replication fork movement [34].

Sustained expression of cyclin E appears to be associated with mitosis rather than meiosis of embryonic cells. Cyclin E expression is translationally repressed during prophase of *C. elegans *gonadal cells [35]. The maintenance of cyclin E expression in these cells actively promotes mitotic division and embryonic gene expression rather than meiosis, and leads to the development of teratomas [35]. A possible explanation for this phenotype is that high cyclin E leads to precocious centrosome assembly, causing exit from meiosis [35].

Cyclin E is also essential in cell fate determination during *Drosophila *neurogenesis, where its expression drives the asymmetric division of neuroblasts into two lineages of glial and neuronal cells [36]. In the absence of cyclin E expression, neuroblasts only produce glial cells rather than the neuronal precursor [36]. This function does not require the interaction of cyclin E with Cdk2, and is mediated via binding and inhibiting the homeobox transcription factor, Prospero [37]. Likewise, in *C. elegans*, cyclin E functions in maintaining stem cell capacity by suppressing the terminal differentiation of quiescent cells, although in combination with Cdk2 [38].

Cyclins E1 and E2 have distinct roles in *Xenopus *development, where cyclin E2, but not cyclin E1, is necessary for viability [39]. This may reflect a dose requirement for E-type cyclins, as their expression in the developing zygote is sequential, with cyclin E1 being maternally expressed from fertilisation to blastula stage, and cyclin E2 expressed at high levels from blastula to tadpole. However, cyclin E1 knockdown at fertilisation does not affect development [39,40] while cyclin E2 knockdown shows a dose dependent effect on viability [39]. Incidentally, the cell cycles prior to the mid blastula transition in *Xenopus *are rapid embryonic cycles where S and M alternate without variation in cyclin E1 levels, whereas the subsequent cycles, with high cyclin E2 expression, have incorporated G_1 _and G_2 _phases [41].

### Knockout mouse models of cyclins E1 and E2

The *Drosophila *and *C. elegans *cyclin E is phylogenetically equidistant to cyclin E1 and cyclin E2 (Figure 1B) and consequently neither cyclin E1 nor cyclin E2 is likely to be the functionally equivalent ortholog to "cyclin E". It has not been established whether cyclin E1 or cyclin E2 are involved in early embryonic cell divisions or asymmetric differentiation in mammals as described above for *Drosophila*, *C. elegans *and *Xenopus*. Mouse embryonic stem cells proliferate in the absence of cyclin E1 or E2 [13,20], so perhaps an E-cyclin is required for the post-embryonic cell cycles, but cyclin A can substitute effectively in embryonic cell cycles which lack p21^Waf1/Cip1 ^and p27^Kip1 ^[20]. These questions can be most effectively addressed by performing cyclin E1 knockin to the cyclin E2 locus, and vice versa.

The phenotype of single knockout mice has yielded clues about E-cyclin function in endoreplication and meiosis (Table 1). Double knockout cyclin E1^-/- ^E2^-/- ^mice die *in utero *due to impairments in endoreplication of the trophoblast giant cells (TSCs) that form the placenta, and perform a crucial role in placental attachment and provision of nutrients to the developing embryo. TSCs normally undergo multiple rounds of DNA replication without mitosis that increase their DNA content to 1000N. TSCs of cyclin E1^-/- ^E2^-/- ^mice barely reach a ploidy of 8N, even with prolonged culture, although they still increase in size and express markers of differentiation consistent with TSC development [20]. Conversely, in Fbw7 knockout mice, which fail to degrade cyclin E1, high cyclin E1 expression is associated with increased DNA synthesis in TSC cells [42]. Another polyploid cell type, the megakaryocyte, similarly fails to reduplicate DNA in cyclin E1^-/- ^E2^-/- ^knockout mice [13]. In the initial studies of this phenomenon no abnormal phenotype of polyploid tissues was detected in single E-cyclin knockout mice [13,20]. However, the E-cyclins are differentially regulated during TSC endoreplication, with cyclin E1 levels declining while cyclin E2 levels remain steady [20]. Cyclin E2 mRNA is also more highly expressed in the polyploid cells of the liver, hepatocytes, where it is found at higher levels in hepatocytes with 8N DNA content than those with 4N DNA content [43]. Together these data suggest that cyclin E1 and cyclin E2 levels may have different roles in endoreplication.

**Table 1:** Attributes of the E-cyclins

	Cyclin E1 (CCNE1)	Cyclin E2 (CCNE2)
**Chromosomal location**	19q12	8q22.1

**Isoforms**	2 (Full length and 15 amino acid N-terminal truncation)	1

**Transcriptional regulation**	*Upregulated by:*	*Upregulated by:*
	E2F1, E2F2, E2F3 [55-57]	E2F1, E2F2, E2F3 [55-57]
	P300/CBP [58], Src3 [59], Carm1 [60]	P300/CBP*, Src3 [61], Carm1 [61]
	*Suppressed by:*	Chd8 [74,75]
	HDAC1 [58,62,63], SUV39H1/HP1 [64,65], BRG1/hBRM [66], PRMT5/COPR5 [68-71]	CDP/Cux p110 [47]
		*Suppressed by:*
		HDAC1*, SUV39H1/HP1*, BRG1/hBRM*, PRMT5/COPR5 [68-71]

**miRNA regulation**	miR15b [86], miRNA 16 family [87]	miR-9, miR-34c and miR200a [88], miR34a [89], miR26a [90]

**Post-translational cleavage**	Yes [98]	No [99]

**Tissue expression**	*Embryonic cells*	*Embryonic cells*
	- Very high in mouse embryonic stem cells [45]	- Absent in mouse embryonic stem cells [45]
	- Sole E-cyclin from fertilisation to blastula stage in *Xenopus *[39]	- Not expressed during embryonic cycles of *Xenopus *[39]
	*Adult tissues*	*Adult tissues*
	- Mouse: moderate in brain, testes and thymus, low in intestine and spleen [45]	- Mouse: high in testes, low to moderate in brain, intestine, muscle and thymus [45]
	- Human: very high in placenta, high in testes, low to moderate in thymus, small intestine and colon [5]	-Human: high in brain, placenta, testes and thymus, low to moderate in spleen, thymus, small intestine and colon [5]
	*Polyploid cells*	*Polyploid cells*
	- Low expression in mature trophoblast giant cells [20]	- Sustained expression in mature trophoblast giant cells [20]
	- Low expression compared to CCNE2 in hepatocytes [44]	- High expression compared to CCNE1 in hepatocytes [44], increased expression with increased polyploidy [43]

**Knockout mouse**	Normal fertility [13,20]	Male infertility and testicular atrophy [13,20]
	Slight delay in liver regeneration following partial hepatectomy [44]	Accelerated liver regeneration and increased hepatocyte polyploidy following partial hepatectomy [44]

* by inference from data on CCNE1

A recent study by Nevzorova *et al *using partial hepatectomy of cyclin E1^-/- ^and cyclin E2^-/- ^knockout mice has shed further light on this subject [44]. Partial hepatectomy of mice leads to a rapid expansion of the polyploid hepatocyte population to regenerate the liver. In cyclin E1^-/- ^mice, this regenerative response was slightly delayed, and associated with a compensatory increase in cyclin A-Cdk2 activity [44]. Surprisingly, cyclin E2^-/- ^mice had accelerated liver regeneration and increased DNA synthesis associated with an upregulation of cyclin E1 expression and cyclin E1-Cdk2 activity [44]. Consequently it appears that cyclin E2 normally acts to repress cyclin E1 expression, thus negatively regulating S phase entry in hepatocytes. The ablation of cyclin E2 leads to increased polyploidy, associated with increased cyclin E1-Cdk2 activity [44]. Thus high cyclin E1 may induce endoreplication, whereas cyclin E2 acts as a brake in this process. These results are distinct from those observed in the other polyploid cell types, TSCs and megakaryocytes, where cyclin E1 and E2 single knockout mice were reported to have "normal" phenotypes. However the morphology and Cdk activity of these tissues has not been explicitly reported in single knockout mice [13,20], so it may be that similar substitutions are occurring in these tissues, where cyclin A is functional in the absence of cyclin E1, and cyclin E1 levels are increased in the absence of cyclin E2.

A further unique phenotype of the cyclin E2 knockout mice is testicular atrophy and reduced male fertility, associated with aberrant meiosis [13]. Could this phenotype be due to low cyclin E2 expression increasing cyclin E1 levels as described for the regenerating liver? This seems unlikely, as cyclin E1 is already expressed at high levels in the mouse and human testes [5,45], but only cyclin E2 deletion results in a phenotype [13,20]. In addition, cyclin E1^+/- ^E2^-/- ^mice display more pronounced testicular hypoplasia and male infertility than cyclin E2^-/- ^mice, indicating that it is unlikely that excess cyclin E1 causes this phenotype, as the phenotype is more severe when a cyclin E1 allele is removed [13]. Meiosis in *C. elegans *specifically requires periodic cyclin E expression [35], so there may be a particular role for cyclin E2 in the meiosis-mitosis switch in mammals. The overexpression of a hyperstable form of cyclin E1 that is not periodically degraded does not lead to altered fertility in mice [46], but this has not been examined in the context of cyclin E2. Of interest, cyclin E2, but not cyclin E1, is upregulated by the p110 isoform of the transcription factor CDP/Cux [47] and CDP/Cux knockout mice also suffer from male infertility, although without testicular atrophy [48].

## Transcriptional Regulation of the E-cyclins

The E-cyclins are cell cycle regulated at both the mRNA and protein level, leading to cell cycle phase-specific expression. Cyclin E1 and E2 mRNA (CCNE1 and CCNE2) peak in mid-G_1 _to early S phase in multiple models of mitotic division [4,5,10]. During S phase cyclin E1 is rapidly downregulated via proteosomal degradation (for reviews of cyclin E1 proteosomal degradation see [2,49]). In brief, during early S phase cyclin E1 is phosphorylated at conserved residues via Cdk2 and GSK-3β, leading to recognition and ubiquitination of cyclin E1 by the ubiquitin ligase SCF^Fbw7^, followed by degradation during S phase [2,49]. The turnover of cyclin E2 is reported to be regulated in a similar manner [50] but has been examined in less detail. Cyclin E1 turnover can also be mediated by ubiquitin ligase components Skp2 [51], Parkin [52] and Cul4 [53], which may contribute to late S-phase degradation of cyclin E1. The combination of transcriptional and post-translational regulation results in an intense peak in expression of cyclin E1 in late G_1 _and early S phase of the cell cycle. The activity of cyclin E1-Cdk2 and cyclin E2-Cdk2 complexes is further refined through binding of the Cdk inhibitors p21^Waf1/Cip1 ^and p27^Kip1^, whose expression is also cell cycle regulated.

The cell cycle dependent transcription of the E-cyclins is mediated by E2F transcription factors, which are activated via release from Rb during late G_1 _phase. Rb-deficient cells have high expression of cyclin E1 and cyclin E2 [45,54], likely due to constitutive E2F release, and numerous gene expression array studies have confirmed both CCNE1 and CCNE2 as E2F1, E2F2 and E2F3 target genes [55-57]. E2F proteins interact with multiple co-regulators at the E-cyclin promoters, allowing for the input of mitogenic signals. E2F1 recruits the histone acetylase p300/CBP [58] and the co-activator SRC3 [59] to the CCNE1 promoter. This complex further recruits the protein methyltransferase Carm1/PRMT4 to the CCNE1 and CCNE2 promoters [60], leading to increased transcription of at least the CCNE1 gene [60,61]. CCNE1 transcription is actively inhibited by Rb and the other pocket proteins in G_0 _and G_1 _arrested cells [58,60,62-66] and during late mitosis [67]. Rb, via an interaction with inhibitory E2Fs (E2F4-6) recruits the histone deacetylase HDAC1 [58,62,63], the methylation complex SUV39H1 and HP1 [64,65] and the BRG1/hBRM nucleosome remodeling complex [66] to the CCNE1 promoter, leading to inhibitory deacetylation, methylation and remodelling of the promoter-associated nucleosomes. The methlytransferase PRMT5, via binding partner COPR5 [68,69], also negatively regulates CCNE1 and CCNE2 transcription [70,71], especially during G_0 _[60].

### Differential transcription of cyclin E1 and E2

CCNE1 regulation has been more closely characterised than CCNE2, with an assumption of similar regulation of the CCNE2 gene [3]. However, CCNE2 appears to be inherently more sensitive to induction by E2F transcription factors than CCNE1, with CCNE2 mRNA showing a 1.5-10 fold greater induction than CCNE1 in 3 separate studies [55-57]. The CCNE2 promoter also shows greater enrichment for E2F binding by chromatin immunoprecipitation [72]. Furthermore, overexpression of a mutant E2F that derepresses but does not activate transcription, significantly induces CCNE2 but not CCNE1 [57]. Since E2F activity is a core component of cell cycle progression, this suggests that CCNE2 expression may be more strongly amplified than CCNE1 in each cell cycle. Interestingly, cyclin E2 becomes expressed at high levels in immortalised mouse embryonic fibroblasts derived from E2F1 knockout animals, and is also expressed at high levels in chemically-induced tumours derived from the same mice, without concurrent changes to cyclin E1 expression [73]. Consequently cyclin E2 may be targeted independently of E2F factors, or at least E2F1.

Two instances have been identified where CCNE2, independently of CCNE1, is markedly upregulated by E2F binding partners. Chd8, a chromatin remodelling enzyme, facilitates efficient RNA polymerase II transcript elongation of a subset of genes, including E2F1 targets. While Chd8 can interact with E2F at the promoters of both CCNE1 and CCNE2, its presence leads only to the upregulation of cyclin E2 [74]. Chd8 binds constitutively to the CCNE2 promoter throughout the cell cycle, but it is required for the upregulation of cyclin E2 during estrogen rescue from anti-estrogen induced quiescence [75], and as cells pass through the G_1_/S-phase transition [74]. Rodriguez-Paredes *et al *propose that Chd8 is recruited to all E2F1-dependent genes, but that only those genes with a specific chromatin structure at the 5' region, such as CCNE2, utilise Chd8 to mobilise RNA polymerase II and nucleosomes during transcript elongation [74]. A distinct chromatin structure may explain why CCNE2 is inherently more sensitive to E2F induction than CCNE1 as described above. Another E2F1 co-activator, CDP/Cux p110, [76] also specifically upregulates CCNE2 without alterations to CCNE1 [47], although binding to the CCNE2 promoter was not demonstrated.

Through their independent regulation by transcription factors, cyclin E1 and cyclin E2 are associated with different networks of genes and thus potentially with distinct biological processes. Cyclin E2 is upregulated via Chd8 downstream of cyclin D1 in estrogen-treated cells [75], and has also been identified as downstream of cyclin D1, or cyclin D1-mediated pathways, in other models [77-79]. Cyclin E1 is often expressed at high levels in the absence of increased cyclin D1 [80], and in fact cyclin E1-Cdk2 activity is increased by estrogen in breast cancer cells primarily through disengagement of p21^Waf1/Cip1 ^rather than transcriptional upregulation by cyclin D1 [75]. In this same model, c-Myc is able to induce cell cycle re-entry through the induction of cyclin E1-Cdk2 activity, but without significantly increasing the expression of cyclin E2 [75]. Thus cyclin E1 and cyclin E2 are distinctly regulated downstream of estrogen through the major regulatory proteins, cyclin D1 and c-Myc [75]. This action may not be confined to estrogen, as cyclin E2 is also induced by androgen, likely downstream of cyclin D1 or D3, although this is not independent of cyclin E1 upregulation [81].

The disparate tissue expression pattern of cyclin E1 and cyclin E2 also suggests that there is differential regulation of the E-cyclins [5,45]. For example, cyclin E2 is expressed at high levels in human brain where cyclin E1 is notably absent [5], whereas cyclin E1 expression is consistently higher than cyclin E2 in the thymus [5,45]. The inhibition of cyclin E1 transcription by cyclin E2 identified in hepatocytes may contribute to the differential expression of the E-cyclins in some tissues [44]. We have made a similar observation in the breast cancer cell line MCF-7 that cyclin E2 knockdown leads to an increase in cyclin E1 protein levels, although this is associated with decreased overall proliferation [75]. However, cyclin E2 modulation does not always lead to changes in cyclin E1 expression, for example in smooth muscle cells the cyclin E2 siRNA treatment leads to downregulation of cyclin E1 [82], and cyclin E1 and E2 are co-expressed in other tissues [5,45], and in some tumours [83].

### Post-transcriptional regulation of cyclin E1 and E2

Another layer of complexity is added through the regulation of the E-cyclins by non-coding RNAs. Despite high conservation, the mRNA sequences of CCNE1 and CCNE2 are predicted and validated targets of distinct subsets of microRNAs (miRNAs) [84,85]. For example CCNE1 is targeted by miR15b [86] and the miRNA 16 family [87], and CCNE2 by miR-9, miR-34c, miR-200a [88], and miR34a [89]. miR-26a specifically targets CCNE2 but not CCNE1 [90]. There is an expressed anti-sense transcript which may further modulate CCNE2 expression [91].

miRNAs frequently target gene networks to alter cellular processes such as proliferation, which raises the question why CCNE1 and CCNE2 appear to be targeted as part of discrete regulatory modules if they are functionally redundant proteins. These modules target distinct subsets of cell cycle proteins and may therefore have subtly different effects on proliferation, for example, mir16 co-represses CCNE1, CCND3 and CDC6 [87]. CCNE2 is independently downregulated by the p53-regulated miRNA, miR34a, in colon cancer cells [89]. This may explain why cyclin E2 mRNA and protein, but not cyclin E1, is induced after viral oncoprotein E6 induces degradation of p53 in normal human fibroblasts [5]. Furthermore p53 expression suppresses CCNE2 in prostate cancer cells [92], and the inactivation of p53 in the mammary gland leads to tumours which are high in CCNE2 [93]. Consequently CCNE2 is frequently suppressed downstream of the p53 tumour suppressor gene, linking CCNE2 to a network of p53 activity independently of CCNE1 [89]. The miRNAs that regulate CCNE1 and CCNE2, as well as the transcription factors discussed above, are often altered in tumorigenesis, which may contribute to the distinct associations of CCNE1 and CCNE2 to different cancer types, as described below.

## Associations of cyclin E1 and cyclin E2 with cancer

Cyclin E1 is a well established oncogene, and its overexpression, especially in a hyperstable form, leads to increased incidence of mouse neoplasia [94-96], and increased susceptibility to other oncogenes [96]. Potential oncogenic effects of cyclin E2 have not been examined in mice, except that mouse embryonic fibroblasts from the E-cyclin double knockout mice are not susceptible to transformation [13]. Further evidence from cell culture models indicates that cyclin E2 has similar proliferative effects to cyclin E1 in cancer cells. The overexpression of either cyclin E1 or cyclin E2 leads to a redistribution of cells within cell cycle phases, with a shorter G_1 _[5], and a longer S phase at least in cyclin E1 overexpressing cells [23,31]. The siRNA-mediated decrease of either E-cyclin severely attenuates the estrogen-induced proliferation of breast cancer cells [75], and the reduction of either cyclin E1 or E2 leads to reduced colony forming ability in oral squamous cell carcinoma cells, although cyclin E1 ablation is more potent than ablation of cyclin E2 (70% vs 20% reduction) [97]. Cyclin E1 may have higher potency than cyclin E2 as it can be cleaved into low molecular weight fragments with enhanced oncogenic activity [98], and cyclin E2 does not appear to be similarly processed [99].

While cyclin E1-Cdk2 and cyclin E2-Cdk2 kinase activities are increased in breast cancer compared to normal tissue [83], cyclin E1 and E2 expression and kinase activity are not essential for proliferation of all cancer cell types [97]. In some cases increases in cyclin E2 expression are not associated with proliferation, but instead with other oncogenic attributes such as invasion [100] and drug resistance [101,102]. Cyclin E1 may enhance tumorigenesis through increasing genomic instability, an ability which may be derived from promoting premature replication licensing and endoreplication [96,103]. Specific experiments have not been reported that examine whether genomic instability is also induced through cyclin E2 overexpression. Given that recent evidence seems to identify cyclin E1, but not cyclin E2, as a mediator of endoreplication in hepatocytes [44], cyclin E1 and E2 may not have equivalent roles in the induction of genomic instability.

Due to the limited availability of antibodies suitable for immunohistochemistry, the expression of cyclin E2 has been examined only through mRNA expression in tumour samples, whereas there is a comprehensive array of literature on the expression of both cyclin E1 protein and mRNA in cancer samples [2,104]. CCNE1 and CCNE2 expression has been compared in breast cancer, where these studies are representative of the majority of studies on the relationship of CCNE1 to breast cancer [104]. Both CCNE1 and CCNE2 are expressed at higher levels in breast cancer, with an association of both genes to increased tumour grade [105,106], estrogen receptor (ER) negative status [105,106], progesterone receptor negative status [106], and proliferative index by Ki67 staining [83,105]. High levels of CCNE1 have a more significant relationship than CCNE2 with each of these parameters. Both CCNE1 and CCNE2 have an inverse linear relationship between expression and metastasis-free survival, which is particularly strong for CCNE2 [107], and high expression of each E-cyclin mRNA also predicts poor overall survival [106] and shorter relapse-free interval [105]. One study also found some correlation between the expression of CCNE1 and CCNE2 [83], although each E-cyclin is independently corrleated to poor overall and metastasis-free survival [106]. CCNE2 is also a component of three prognostic gene expression signatures that predict shorter metastasis-free survival or relapse-free survival of breast cancer patients, whereas CCNE1 does not feature in any of these signatures [108-110].

Differences become apparent between CCNE1 and CCNE2 in their relationship to survival and response to therapy of ER positive and anti-estrogen (tamoxifen) treated patients. High CCNE1 expression predicts shorter metastasis-free survival in both ER negative and ER positive patients, whereas CCNE2 expression is only predictive for the ER positive patient subset [105,106]. By contrast, in primary tumours high levels of CCNE1, but not CCNE2, predict a shorter relapse-free interval of tamoxifen-treated patients [105]. However CCNE2 has been detected at high levels in recurrent disease after tamoxifen treatment [111], hinting at a functional, if not prognostic, role. In tamoxifen resistant cell lines, CCNE2, but not CCNE1, is induced as part of the agonist response to tamoxifen [112]. Anti-estrogen resistance in MCF-7 breast cancer cells conferred by the TNFα inhibitor, A20, is also associated with increases in cyclin E2 expression [113]. This is consistent with studies in estrogen-responsive breast cancer cell lines where cyclin E2 is a highly estrogen responsive target, whereas cyclin E1 is only marginally increased [75,114].

CCNE1 is expressed at high levels in multiple tumour types other than breast [2]. CCNE2 shows moderate increases in expression in various malignancies, such as lung, ovarian, nasopharyngeal, colorectal, non small cell lung cancer and leukaemia [83,88,115-117], and these increases in expression are frequently not correlated to CCNE1 expression [83]. In two studies the expression of CCNE2 is lower in ovarian and non small cell lung tumours than matched controls, whereas CCNE1 expression remains unchanged or increases [118,119]. Two further studies indicate that CCNE2 is expressed at significant levels in recurrent disease, including therapy-related myeloid leukemia [120] and recurrent non small cell lung carcinoma [121]. These data reflect the more comprehensive data collected with respect to breast cancer, where CCNE2 expression is frequently upregulated in tumours independently of CCNE1, and CCNE2 is often detected in recurrent disease.

## Concluding remarks

Cyclin E1 and E2 display largely overlapping functions, and high redundancy. Does the presence of two E-cyclins provide a safety net to ensure proliferative potential, or do they have unique functions? The E-cyclin gene pair has been maintained without significant divergence throughout the evolution of higher eukaryotes, implying a selective pressure for the maintenance of each gene. Genome wide studies in yeast have found that apparently redundant paralogs do in fact have distinct and non-overlapping functions, although these only become evident in circumstances of stress or particular stimuli [122]. This is certainly the case for cyclin E1 and E2, where cyclin E1 promotes hepatocyte endoreplication after liver injury, whereas cyclin E2 suppresses this process [44]. Further differences between cyclin E1 and cyclin E2 are possibly found during meiosis and embryogenesis [13,39]. There are tantalising observations on the activity of cyclin E in *Drosophila *and *C. elegans *which suggest that there may be additional functions for the E-cyclins in DNA replication of embryonic cells and lineage determination of stem cells. Consequently the differences between cyclin E1 and E2 may be more apparent in non-mitotic cell cycles than in normally proliferating cells, which may explain their apparent redundancy in common laboratory model systems.

Cancer cell cycles represent another form of aberrant cell division, often compared to embryonic cell cycles, and cyclins E1 and E2 promote oncogenic transformation and to promote cancer cell proliferation [13]. Data from cancer studies have already identified that cyclin E1 and E2 are frequently not co-expressed in tumours, and may have distinct associations with recurrent disease. A likely explanation for the discordant expression of cyclin E1 and E2 in cancer is their regulation by distinct subsets of transcription factors and miRNAs. The expression of cyclin E2, independently of cyclin E1, can be induced via cyclin D1, Chd8 and CDP/Cux, all of which are upregulated in cancers [47,123], and cyclin E2 is also independently suppressed by the tumour suppressor p53 [89]. The co-regulation of cyclin E1 and cyclin E2 with distinct gene sets could explain the different relationship of each gene to disease. For example, cyclin E2, which is a target of cyclin D1 and Chd8 in estrogen-responsive cells [75], has been associated with poor outcome only in ER-positive breast cancers [106], which resembles the association of cyclin D1 overexpression to ER-positive but not ER-negative breast cancers [124]. The functional outcome of overexpression of cyclin E1 compared to cyclin E2 is not known. However, given the potential differences in function in endoreplication and other processes, cyclin E1 and cyclin E2 may have distinct attributes that contribute to cancer progression.

The cyclin and Cdk proteins of the cell cycle have considerable redundancy and compensation between members of each family [125], yet careful study reveals unique functions of many of these proteins. For example, cyclin D2 knockin cannot completely substitute for cyclin D1 in mouse development [126]. Recent data on the function and regulation of cyclin E1 and cyclin E2 demonstrates that they are also not redundant homologs (Table 1). The identification of cyclin E1 as promoting, and cyclin E2 as repressing, hepatocyte endoreplication deserves further investigation to unravel the mechanistic difference between the two proteins. Cyclin E1 and E2 should also be investigated as distinct entities in cancer studies, especially in the context of examining relationships with other markers of tumorigenesis such as cyclin D1 and p53. There will be difficulties in ongoing studies on cyclin E1 and cyclin E2 as they do have considerable functional redundancy in their interactions with Cdk2 and Cdk inhibitor proteins, as well as having the potential to regulate one another. The identification of further differences between the E-cyclins is likely to require an appropriate molecular environment that highlights their differences, such as during endoreplication or the proliferation of cancer cells.

## Abbreviations

CCNE1: Cyclin E1 mRNA; CCNE2: Cyclin E2 mRNA; Cdk: cyclin dependent kinase; CLS: centrosome localisation sequence; ER: estrogen receptor; miRNA: microRNA; Pre-RC: pre replication complex; TSC: trophoblast giant cell.

## Competing interests

The authors declare that they have no competing interests.

## Authors' contributions

CC and EM drafted the manuscript. Both authors read and approved the final manuscript.

## References

[B1] Koff A Cross F Fisher A Schumacher J Leguellec K Philippe M Roberts JM Human cyclin E, a new cyclin that interacts with two members of the CDC2 gene family Cell 1991 66 1217 1228 10.1016/0092-8674(91)90044-Y 1833068

[B2] Hwang HC Clurman BE Cyclin E in normal and neoplastic cell cycles Oncogene 2005 24 2776 2786 10.1038/sj.onc.1208613 15838514

[B3] Möröy T Geisen C Cyclin E The International Journal of Biochemistry & Cell Biology 2004 36 1424 1439 10.1016/j.biocel.2003.12.005 15147722

[B4] Lauper N Beck AR Cariou S Richman L Hofmann K Reith W Slingerland JM Amati B Cyclin E2: a novel CDK2 partner in the late G_1 _and S phases of the mammalian cell cycle Oncogene 1998 17 2637 2643 10.1038/sj.onc.1202477 9840927

[B5] Zariwala M Liu J Xiong Y Cyclin E2, a novel human G_1 _cyclin and activating partner of CDK2 and CDK3, is induced by viral oncoproteins Oncogene 1998 17 2787 2798 10.1038/sj.onc.1202505 9840943

[B6] Su TT Stumpff J Promiscuity rules? The dispensability of cyclin E and Cdk2 Sci STKE 2004 2004 pe11 10.1126/stke.2242004pe11 15026579 PMC3242733

[B7] Malumbres M Barbacid M Mammalian cyclin-dependent kinases Trends Biochem Sci 2005 30 630 641 10.1016/j.tibs.2005.09.005 16236519

[B8] Hochegger H Takeda S Hunt T Cyclin-dependent kinases and cell-cycle transitions: does one fit all? Nat Rev Mol Cell Biol 2008 9 910 916 10.1038/nrm2510 18813291

[B9] Harper JW Adams PD Cyclin-Dependent Kinases Chemical Reviews 2001 101 2511 2526 10.1021/cr0001030 11749386

[B10] Gudas JM Payton M Thukral S Chen E Bass M Robinson MO Coats S Cyclin E2, a novel G_1 _cyclin that binds Cdk2 and is aberrantly expressed in human cancers Mol Cell Biol 1999 19 612 622 9858585 10.1128/mcb.19.1.612 PMC83919

[B11] Aleem E Kiyokawa H Kaldis P Cdc2-cyclin E complexes regulate the G_1_/S phase transition Nat Cell Biol 2005 7 831 836 10.1038/ncb1284 16007079

[B12] Geisen C Möröy T The oncogenic activity of cyclin E Is not confined to Cdk2 activation alone but relies on several other, distinct functions of the protein J Biol Chem 2002 277 39909 39918 10.1074/jbc.M205919200 12149264

[B13] Geng Y Yu Q Sicinska E Das M Schneider JE Bhattacharya S Rideout WM Bronson RT Gardner H Sicinski P Cyclin E ablation in the mouse Cell 2003 114 431 443 10.1016/S0092-8674(03)00645-7 12941272

[B14] Karsunky H Geisen C Schmidt T Haas K Zevnik B Gau E Moroy T Oncogenic potential of cyclin E in T-cell lymphomagenesis in transgenic mice: evidence for cooperation between cyclin E and Ras but not Myc Oncogene 1999 18 7816 7824 10.1038/sj.onc.1203205 10618723

[B15] Lukas J Herzinger T Hansen K Moroni MC Resnitzky D Helin K Reed SI Bartek J Cyclin E-induced S phase without activation of the pRb/E2F pathway Genes Dev 1997 11 1479 1492 10.1101/gad.11.11.1479 9192874

[B16] Sweeney KJ Swarbrick A Sutherland RL Musgrove EA Lack of relationship between CDK activity and G_1 _cyclin expression in breast cancer cells Oncogene 1998 16 2865 2878 10.1038/sj.onc.1201814 9671407

[B17] Blow JJ Gillespie PJ Replication licensing and cancer - a fatal entanglement? Nat Rev Cancer 2008 8 799 806 10.1038/nrc2500 18756287 PMC2577763

[B18] Geng Y Lee Y-M Welcker M Swanger J Zagozdzon A Winer JD Roberts JM Kaldis P Clurman BE Sicinski P Kinase-independent function of cyclin E Mol Cell 2007 25 127 139 10.1016/j.molcel.2006.11.029 17218276

[B19] Lee HO Davidson JM Duronio RJ Endoreplication: polyploidy with purpose Genes Dev 2009 23 2461 2477 10.1101/gad.1829209 19884253 PMC2779750

[B20] Parisi T Beck AR Rougier N McNeil T Lucian L Werb Z Amati B Cyclins E1 and E2 are required for endoreplication in placental trophoblast giant cells EMBO J 2003 22 4794 4803 10.1093/emboj/cdg482 12970191 PMC212738

[B21] Su TT O' Farrell PH Chromosome association of minichromosome maintenance proteins in *Drosophila *endoreplication cycles J Cell Biol 1998 140 451 460 10.1083/jcb.140.3.451 9456309 PMC2140170

[B22] Matsumoto Y Maller JL A centrosomal localization signal in cyclin E required for Cdk2-independent S phase entry Science 2004 306 885 888 10.1126/science.1103544 15514162

[B23] Ekholm-Reed S Mendez J Tedesco D Zetterberg A Stillman B Reed SI Deregulation of cyclin E in human cells interferes with prereplication complex assembly J Cell Biol 2004 165 789 800 10.1083/jcb.200404092 15197178 PMC2172392

[B24] Ferguson RL Maller JL Cyclin E-dependent localization of MCM5 regulates centrosome duplication J Cell Sci 2008 121 3224 3232 10.1242/jcs.034702 18799789

[B25] Fay D Han M Mutations in cye-1, a *Caenorhabditis elegans *cyclin E homolog, reveal coordination between cell-cycle control and vulval development Development 2000 127 4049 4060 10952902 10.1242/dev.127.18.4049

[B26] Knoblich JA Sauer K Jones L Richardson H Saint R Lehner CF Cyclin E controls S phase progression and its down-regulation during *Drosophila *embryogenesis is required for the arrest of cell proliferation Cell 1994 77 107 120 10.1016/0092-8674(94)90239-9 8156587

[B27] Lilly MA Spradling AC The *Drosophila *endocycle is controlled by cyclin E and lacks a checkpoint ensuring S-phase completion Genes Dev 1996 10 2514 2526 10.1101/gad.10.19.2514 8843202

[B28] Furstenthal L Kaiser BK Swanson C Jackson PK Cyclin E uses Cdc6 as a chromatin-associated receptor required for DNA replication J Cell Biol 2001 152 1267 1278 10.1083/jcb.152.6.1267 11257126 PMC2199215

[B29] Richardson H O' Keefe L Reed S Saint R A *Drosophila *G1-specific cyclin E homolog exhibits different modes of expression during embryogenesis Development 1993 119 673 690 8187637 10.1242/dev.119.3.673

[B30] Rempel RE Sleight SB Maller JL Maternal *Xenopus *Cdk2-cyclin E complexes function during meiotic and early embryonic cell cycles that lack a G_1 _phase J Biol Chem 1995 270 6843 6855 10.1074/jbc.270.28.16918 7896832

[B31] Keck JM Summers MK Tedesco D Ekholm-Reed S Chuang L-C Jackson PK Reed SI Cyclin E overexpression impairs progression through mitosis by inhibiting APC^Cdh1^ J Cell Biol 2007 178 371 385 10.1083/jcb.200703202 17664332 PMC2064850

[B32] Schnackenberg BJ Marzluff WF Novel localization and possible functions of cyclin E in early sea urchin development J Cell Sci 2002 115 113 121 11801729 10.1242/jcs.115.1.113

[B33] Park EA MacAlpine DM Orr-Weaver TL *Drosophila *follicle cell amplicons as models for metazoan DNA replication: A cyclinE mutant exhibits increased replication fork elongation Proc Natl Acad Sci USA 2007 104 16739 16746 10.1073/pnas.0707804104 17940024 PMC2040429

[B34] Alexandrow MG Hamlin JL Chromatin decondensation in S-phase involves recruitment of Cdk2 by Cdc45 and histone H1 phosphorylation J Cell Biol 2005 168 875 886 10.1083/jcb.200409055 15753125 PMC2171796

[B35] Biedermann B Wright J Senften M Kalchhauser I Sarathy G Lee M-H Ciosk R Translational repression of *cyclin E *prevents precocious mitosis and embryonic gene activation during *C. elegans *meiosis Dev Cell 2009 17 355 364 10.1016/j.devcel.2009.08.003 19758560

[B36] Berger C Pallavi SK Prasad M Shashidhara LS Technau GM A critical role for cyclin E in cell fate determination in the central nervous system of *Drosophila melanogaster* Nat Cell Biol 2005 7 56 62 10.1038/ncb1203 15580266

[B37] Berger C Kannan R Myneni S Renner S Shashidhara LS Technau GM Cell cycle independent role of cyclin E during neural cell fate specification in *Drosophila *is mediated by its regulation of Prospero function Dev Biol 2009 12 12 10.1016/j.ydbio.2009.11.012 19914234

[B38] Fujita M Takeshita H Sawa H Cyclin E and CDK2 repress the terminal differentiation of quiescent cells after asymmetric division in *C. elegans* PLoS ONE 2007 2 e407 10.1371/journal.pone.0000407 17476329 PMC1852333

[B39] Gotoh T Shigemoto N Kishimoto T Cyclin E2 is required for embryogenesis in *Xenopus laevis* Dev Biol 2007 310 341 347 10.1016/j.ydbio.2007.08.005 17825278

[B40] Slevin MK Lyons-Levy G Weeks DL Hartley RS Antisense knockdown of cyclin E does not affect the midblastula transition in *Xenopus laevis *embryos Cell Cycle 2005 4 1396 1402 16131839 10.4161/cc.4.10.2035

[B41] Howe JA Newport JW A developmental timer regulates degradation of cyclin E1 at the midblastula transition during *Xenopus *embryogenesis Proc Natl Acad Sci USA 1996 93 2060 2064 10.1073/pnas.93.5.2060 8700885 PMC39909

[B42] Tetzlaff MT Yu W Li M Zhang P Finegold M Mahon K Harper JW Schwartz RJ Elledge SJ Defective cardiovascular development and elevated cyclin E and Notch proteins in mice lacking the Fbw7 F-box protein Proc Natl Acad Sci USA 2004 101 3338 3345 10.1073/pnas.0307875101 14766969 PMC373463

[B43] Lu P Prost S Caldwell H Tugwood JD Betton GR Harrison DJ Microarray analysis of gene expression of mouse hepatocytes of different ploidy Mamm Genome 2007 18 617 626 10.1007/s00335-007-9048-y 17726633

[B44] Nevzorova YA Tschaharganeh D Gassler N Geng Y Weiskirchen R Sicinski P Trautwein C Liedtke C Aberrant cell cycle progression and endoreplication in regenerating livers of mice that lack a single E-type cyclin Gastroenterology 2009 137 691 703 e696 10.1053/j.gastro.2009.05.003 19445941 PMC2730664

[B45] Geng Y Yu Q Whoriskey W Dick F Tsai KY Ford HL Biswas DK Pardee AB Amati B Jacks T Richardson A Dyson N Sicinski P Expression of cyclins E1 and E2 during mouse development and in neoplasia Proc Natl Acad Sci USA 2001 98 13138 13143 10.1073/pnas.231487798 11687642 PMC60837

[B46] Minella AC Loeb KR Knecht A Welcker M Varnum-Finney BJ Bernstein ID Roberts JM Clurman BE Cyclin E phosphorylation regulates cell proliferation in hematopoietic and epithelial lineages *in vivo* Genes Dev 2008 22 1677 1689 10.1101/gad.1650208 18559482 PMC2428064

[B47] Sansregret L Goulet B Harada R Wilson B Leduy L Bertoglio J Nepveu A The p110 isoform of the CDP/Cux transcription factor accelerates entry into S phase Mol Cell Biol 2006 26 2441 2455 10.1128/MCB.26.6.2441-2455.2006 16508018 PMC1430290

[B48] Luong MX Meijden CM van der Xing D Hesselton R Monuki ES Jones SN Lian JB Stein JL Stein GS Neufeld EJ van Wijnen AJ Genetic ablation of the CDP/Cux protein C terminus results in hair cycle defects and reduced male fertility Mol Cell Biol 2002 22 1424 1437 10.1128/MCB.22.5.1424-1437.2002 11839809 PMC134686

[B49] Welcker M Clurman BE FBW7 ubiquitin ligase: a tumour suppressor at the crossroads of cell division, growth and differentiation Nat Rev Cancer 2008 8 83 93 10.1038/nrc2290 18094723

[B50] Klotz K Cepeda D Tan Y Sun D Sangfelt O Spruck C SCF^Fbxw7/hCdc4 ^targets cyclin E2 for ubiquitin-dependent proteolysis Exp Cell Res 2009 315 1832 1839 10.1016/j.yexcr.2008.11.017 19084516

[B51] Hu R Aplin AE Skp2 regulates G_2_/M progression in a p53-dependent manner Mol Biol Cell 2008 19 4602 4610 10.1091/mbc.E07-11-1137 18716061 PMC2575176

[B52] Ikeuchi K Marusawa H Fujiwara M Matsumoto Y Endo Y Watanabe T Iwai A Sakai Y Takahashi R Chiba T Attenuation of proteolysis-mediated cyclin E regulation by alternatively spliced *Parkin *in human colorectal cancers Int J Cancer 2009 125 2029 2035 10.1002/ijc.24565 19585504

[B53] Zou Y Mi J Cui J Lu D Zhang X Guo C Gao G Liu Q Chen B Shao C Gong Y Characterization of nuclear localization signal in N-terminus of CUL4B and its essential role in cyclin E degradation and cell cycle progression J Biol Chem 2009 10.1074/jbc.M1109.050427 PMC2785175 19801544

[B54] Liu H Knabb JR Spike BT Macleod KF Elevated poly-(ADP-ribose)-polymerase activity sensitizes retinoblastoma-deficient cells to DNA damage-induced necrosis Mol Cancer Res 2009 7 1099 1109 10.1158/1541-7786.MCR-08-0439 19584263 PMC2994938

[B55] Stanelle J Stiewe T Theseling CC Peter M Putzer BM Gene expression changes in response to E2F1 activation Nucleic Acids Res 2002 30 1859 1867 10.1093/nar/30.8.1859 11937641 PMC113199

[B56] Muller H Bracken AP Vernell R Moroni MC Christians F Grassilli E Prosperini E Vigo E Oliner JD Helin K E2Fs regulate the expression of genes involved in differentiation, development, proliferation, and apoptosis Genes Dev 2001 15 267 285 10.1101/gad.864201 11159908 PMC312619

[B57] Young AP Nagarajan R Longmore GD Mechanisms of transcriptional regulation by Rb-E2F segregate by biological pathway Oncogene 2003 22 7209 7217 10.1038/sj.onc.1206804 14562049

[B58] Bandyopadhyay D Okan NA Bales E Nascimento L Cole PA Medrano EE Down-regulation of p300/CBP histone acetyltransferase activates a senescence checkpoint in human melanocytes Cancer Res 2002 62 6231 6239 12414652

[B59] Louie MC Zou JX Rabinovich A Chen H-W ACTR/AIB1 functions as an E2F1 coactivator to promote breast cancer cell proliferation and antiestrogen resistance Mol Cell Biol 2004 24 5157 5171 10.1128/MCB.24.12.5157-5171.2004 15169882 PMC419858

[B60] El Messaoudi S Fabbrizio E Rodriguez C Chuchana P Fauquier L Cheng D Theillet C Vandel L Bedford MT Sardet C Coactivator-associated arginine methyltransferase 1 (CARM1) is a positive regulator of the *cyclin E1 *gene Proc Natl Acad Sci USA 2006 103 13351 13356 10.1073/pnas.0605692103 16938873 PMC1569167

[B61] Frietze S Lupien M Silver PA Brown M CARM1 regulates estrogen-stimulated breast cancer growth through up-regulation of E2F1 Cancer Res 2008 68 301 306 10.1158/0008-5472.CAN-07-1983 18172323

[B62] Brehm A Miska EA McCance DJ Reid JL Bannister AJ Kouzarides T Retinoblastoma protein recruits histone deacetylase to repress transcription Nature 1998 391 597 601 10.1038/35404 9468139

[B63] Morrison AJ Sardet C Herrera RE Retinoblastoma protein transcriptional repression through histone deacetylation of a single nucleosome Mol Cell Biol 2002 22 856 865 10.1128/MCB.22.3.856-865.2002 11784861 PMC133558

[B64] Nielsen SJ Schneider R Bauer U-M Bannister AJ Morrison A O' Carroll D Firestein R Cleary M Jenuwein T Herrera RE Kouzarides T Rb targets histone H3 methylation and HP1 to promoters Nature 2001 412 561 565 10.1038/35087620 11484059

[B65] Vandel L Nicolas E Vaute O Ferreira R Ait-Si-Ali S Trouche D Transcriptional repression by the Retinoblastoma protein through the recruitment of a histone methyltransferase Mol Cell Biol 2001 21 6484 6494 10.1128/MCB.21.19.6484-6494.2001 11533237 PMC99795

[B66] Zhang HS Gavin M Dahiya A Postigo AA Ma D Luo RX Harbour JW Dean DC Exit from G_1 _and S phase of the cell cycle is regulated by repressor complexes containing HDAC-Rb-hSWI/SNF and Rb-hSWI/SNF Cell 2000 101 79 89 10.1016/S0092-8674(00)80625-X 10778858

[B67] Polanowska J Fabbrizio E Le Cam L Trouche D Emiliani S Herrera R Sardet C The periodic down regulation of *cyclin E *gene expression from exit of mitosis to end of G_1 _is controlled by a deacetylase- and E2F-associated bipartite repressor element Oncogene 2001 20 4115 4127 10.1038/sj.onc.1204514 11464278

[B68] Lacroix M Messaoudi SE Rodier G Le Cam A Sardet C Fabbrizio E The histone-binding protein COPR5 is required for nuclear functions of the protein arginine methyltransferase PRMT5 EMBO Rep 2008 9 452 458 10.1038/embor.2008.45 18404153 PMC2373370

[B69] Fabbrizio E El Messaoudi S Polanowska J Paul C Cook JR Lee JH Negre V Rousset M Pestka S Le Cam A Sardet C Negative regulation of transcription by the type II arginine methyltransferase PRMT5 EMBO Rep 2002 3 641 645 10.1093/embo-reports/kvf136 12101096 PMC1084190

[B70] Pal S Vishwanath SN Erdjument-Bromage H Tempst P Sif S Human SWI/SNF-associated PRMT5 methylates histone H3 arginine 8 and negatively regulates expression of ST7 and NM23 tumor suppressor genes Mol Cell Biol 2004 24 9630 9645 10.1128/MCB.24.21.9630-9645.2004 15485929 PMC522266

[B71] Teng Y Girvan AC Casson LK Pierce WM Jr Qian M Thomas SD Bates PJ AS1411 alters the localization of a complex containing Protein Arginine Methyltransferase 5 and Nucleolin Cancer Res 2007 67 10491 10500 10.1158/0008-5472.CAN-06-4206 17974993

[B72] Bieda M Xu X Singer MA Green R Farnham PJ Unbiased location analysis of E2F1-binding sites suggests a widespread role for E2F1 in the human genome Genome Res 2006 16 595 605 10.1101/gr.4887606 16606705 PMC1457046

[B73] Ishii H Mimori K Yoshikawa Y Mori M Furukawa Y Vecchione A Differential roles of E-type cyclins during transformation of murine E2F-1-deficient cells DNA Cell Biol 2005 24 173 179 10.1089/dna.2005.24.173 15767783

[B74] Rodriguez-Paredes M Ceballos-Chavez M Esteller M Garcia-Dominguez M Reyes JC The chromatin remodeling factor CHD8 interacts with elongating RNA polymerase II and controls expression of the cyclin E2 gene Nucleic Acids Res 2009 37 2449 2460 10.1093/nar/gkp101 19255092 PMC2677868

[B75] Caldon CE Sergio CM Schutte J Boersma MN Sutherland RL Carroll JS Musgrove EA Estrogen regulation of cyclin E2 requires cyclin D1, but not c-Myc Mol Cell Biol 2009 29 4623 4639 10.1128/MCB.00269-09 19564413 PMC2725719

[B76] Truscott M Harada R Vadnais C Robert F Nepveu A p110 CUX1 cooperates with E2F transcription factors in the transcriptional activation of cell cycle-regulated genes Mol Cell Biol 2008 28 3127 3138 10.1128/MCB.02089-07 18347061 PMC2423173

[B77] Bruno RD Gover TD Burger AM Brodie AM Njar VCO 17α-Hydroxylase/17,20 lyase inhibitor VN/124-1 inhibits growth of androgen-independent prostate cancer cells via induction of the endoplasmic reticulum stress response Mol Cancer Ther 2008 7 2828 2836 10.1158/1535-7163.MCT-08-0336 18790763 PMC4040979

[B78] Eskandarpour M Huang F Reeves KA Clark E Hansson J Oncogenic *NRAS *has multiple effects on the malignant phenotype of human melanoma cells cultured *in vitro* Int J Cancer 2009 124 16 26 10.1002/ijc.23876 18814281

[B79] Wu Z Cho H Hampton GM Theodorescu D Cdc6 and cyclin E2 are PTEN-regulated genes associated with human prostate cancer metastasis Neoplasia 2009 11 66 76 19107233 10.1593/neo.81048 PMC2606120

[B80] Loden M Stighall M Nielsen NH Roos G Emdin SO Ostlund H Landberg G The cyclin D1 high and cyclin E high subgroups of breast cancer: separate pathways in tumorigenesis based on pattern of genetic aberrations and inactivation of the pRb node Oncogene 2002 21 4680 4690 10.1038/sj.onc.1205578 12096344

[B81] Xu Y Chen S-Y Ross KN Balk SP Androgens induce prostate cancer cell proliferation through Mammalian Target of Rapamycin activation and post-transcriptional increases in cyclin D proteins Cancer Res 2006 66 7783 7792 10.1158/0008-5472.CAN-05-4472 16885382

[B82] Dapas B Farra R Grassi M Giansante C Fiotti N Uxa L Rainaldi G Mercatanti A Colombatti A Spessotto P Lacovich V Guarnieri G Grassi G Role of E2F1-cyclin E1-cyclin E2 circuit in human coronary smooth muscle cell proliferation and therapeutic potential of its downregulation by siRNAs Mol Med 2009 15 297 306 10.2119/molmed.2009.00030 19603101 PMC2710289

[B83] Payton M Scully S Chung G Coats S Deregulation of cyclin E2 expression and associated kinase activity in primary breast tumors Oncogene 2002 21 8529 8534 10.1038/sj.onc.1206035 12466974

[B84] TargetScan Human: Prediction of miRNA targets http://www.targetscan.org

[B85] Lewis BP Burge CB Bartel DP Conserved seed pairing, often flanked by adenosines, indicates that thousands of human genes are microRNA targets Cell 2005 120 15 20 10.1016/j.cell.2004.12.035 15652477

[B86] Xia H Qi Y Ng SS Chen X Chen S Fang M Li D Zhao Y Ge R Li G Chen Y He M-L Kung H-f Lai L Lin MC MicroRNA-15b regulates cell cycle progression by targeting cyclins in glioma cells Biochem Biophys Res Commun 2009 380 205 210 10.1016/j.bbrc.2008.12.169 19135980

[B87] Liu Q Fu H Sun F Zhang H Tie Y Zhu J Xing R Sun Z Zheng X miR-16 family induces cell cycle arrest by regulating multiple cell cycle genes Nucleic Acids Res 2008 36 5391 5404 10.1093/nar/gkn522 18701644 PMC2532718

[B88] Chen HC Chen GH Chen YH Liao WL Liu CY Chang KP Chang YS Chen SJ MicroRNA deregulation and pathway alterations in nasopharyngeal carcinoma Br J Cancer 2009 100 1002 1011 10.1038/sj.bjc.6604948 19293812 PMC2661776

[B89] He L He X Lim LP de Stanchina E Xuan Z Liang Y Xue W Zender L Magnus J Ridzon D Jackson AL Linsley PS Chen C Lowe SW Cleary MA Hannon GJ A microRNA component of the p53 tumour suppressor network Nature 2007 447 1130 1134 10.1038/nature05939 17554337 PMC4590999

[B90] Kota J Chivukula RR O'Donnell KA Wentzel EA Montgomery CL Hwang H-W Chang T-C Vivekanandan P Torbenson M Clark KR Mendell JR Mendell JT Therapeutic microRNA delivery suppresses tumorigenesis in a murine liver cancer model Cell 2009 137 1005 1017 10.1016/j.cell.2009.04.021 19524505 PMC2722880

[B91] Yelin R Dahary D Sorek R Levanon EY Goldstein O Shoshan A Diber A Biton S Tamir Y Khosravi R Nemzer S Pinner E Walach S Bernstein J Savitsky K Rotman G Widespread occurrence of antisense transcription in the human genome Nat Biotechnol 2003 21 379 386 10.1038/nbt808 12640466

[B92] Spurgers KB Gold DL Coombes KR Bohnenstiehl NL Mullins B Meyn RE Logothetis CJ McDonnell TJ Identification of cell cycle regulatory genes as principal targets of p53-mediated transcriptional repression J Biol Chem 2006 281 25134 25142 10.1074/jbc.M513901200 16798743

[B93] Lin S-CJ Lee K-F Nikitin AY Hilsenbeck SG Cardiff RD Li A Kang K-W Frank SA Lee W-H Lee EY-HP Somatic mutation of p53 leads to estrogen receptor α-positive and -negative mouse mammary tumors with high frequency of metastasis Cancer Res 2004 64 3525 3532 10.1158/0008-5472.CAN-03-3524 15150107

[B94] Bortner D Rosenberg M Induction of mammary gland hyperplasia and carcinomas in transgenic mice expressing human cyclin E Molecular and Cellular Biology 1997 17 453 459 8972226 10.1128/mcb.17.1.453 PMC231770

[B95] Ma Y Fiering S Black C Liu X Yuan Z Memoli VA Robbins DJ Bentley HA Tsongalis GJ Demidenko E Freemantle SJ Dmitrovsky E Transgenic cyclin E triggers dysplasia and multiple pulmonary adenocarcinomas Proc Natl Acad Sci USA 2007 104 4089 4094 10.1073/pnas.0606537104 17360482 PMC1820713

[B96] Loeb KR Kostner H Firpo E Norwood T D Tsuchiya K Clurman BE Roberts JM A mouse model for cyclin E-dependent genetic instability and tumorigenesis Cancer Cell 2005 8 35 47 10.1016/j.ccr.2005.06.010 16023597

[B97] Yamada S Sumrejkanchanakij P Amagasa T Ikeda M-A Loss of cyclin E requirement in cell growth of an oral squamous cell carcinoma cell line implies deregulation of its downstream pathway Int J Cancer 2004 111 17 22 10.1002/ijc.20234 15185338

[B98] Wingate H Puskas A Duong M Bui T Richardson D Liu Y Tucker SL Van Pelt C Meijer L Hunt K Keyomarsi K Low molecular weight cyclin E is specific in breast cancer and is associated with mechanisms of tumor progression Cell Cycle 2009 8 1062 1068 19305161 10.4161/cc.8.7.8119 PMC2692060

[B99] Guo X Hartley RS HuR contributes to cyclin E1 deregulation in MCF-7 breast cancer cells Cancer Res 2006 66 7948 7956 10.1158/0008-5472.CAN-05-4362 16912169

[B100] Wang J Chen S Screening and identification of gastric adenocarcinoma metastasis-related genes using cDNA microarray coupled to FDD-PCR J Cancer Res Clin Oncol 2002 128 547 553 10.1007/s00432-002-0379-5 12384798 PMC12164413

[B101] de Angelis PM Fjell B Kravik KL Haug T Tunheim SH Reichelt W Beigi M Clausen OP Galteland E Stokke T Molecular characterizations of derivatives of HCT116 colorectal cancer cells that are resistant to the chemotherapeutic agent 5-fluorouracil Int J Oncol 2004 24 1279 1288 15067352

[B102] Elmore LW Di X Dumur C Holt SE Gewirtz DA Evasion of a single-step, chemotherapy-induced senescence in breast cancer cells: implications for treatment response Clin Cancer Res 2005 11 2637 2643 10.1158/1078-0432.CCR-04-1462 15814644

[B103] Spruck CH Won KA Reed SI Deregulated cyclin E induces chromosome instability Nature 1999 401 297 300 10.1038/45836 10499591

[B104] Wang L Shao Z-M Cyclin E expression and prognosis in breast cancer patients: A meta-analysis of published studies Cancer Invest 2006 24 581 587 10.1080/07357900600894799 16982462

[B105] Desmedt C Ouriaghli FE Durbecq V Soree A Colozza MA Azambuja E Paesmans M Larsimont D Buyse M Harris A Piccart M Martiat P Sotiriou C Impact of cyclins E, neutrophil elastase and proteinase 3 expression levels on clinical outcome in primary breast cancer patients Int J Cancer 2006 119 2539 2545 10.1002/ijc.22149 16929516

[B106] Sieuwerts AM Look MP Meijer-van Gelder ME Timmermans M Trapman AM Garcia RR Arnold M Goedheer AJ de Weerd V Portengen H Klijn JG Foekens JA Which cyclin E prevails as prognostic marker for breast cancer? Results from a retrospective study involving 635 lymph node-negative breast cancer patients Clin Cancer Res 2006 12 3319 3328 10.1158/1078-0432.CCR-06-0225 16740753

[B107] Kreike B Hart G Bartelink H Vijver M van de Analysis of breast cancer related gene expression using natural splines and the Cox proportional hazard model to identify prognostic associations Breast Cancer Res Treat 2009 in press 10.1007/s10549-009-0588-6 19859804

[B108] Sotiriou C Wirapati P Loi S Harris A Fox S Smeds J Nordgren H Farmer P Praz V Haibe-Kains B Desmedt C Larsimont D Cardoso F Peterse H Nuyten D Buyse M Vijver MJ Van de Bergh J Piccart M Delorenzi M Gene expression profiling in breast cancer: understanding the molecular basis of histologic grade to improve prognosis J Natl Cancer Inst 2006 98 262 272 16478745 10.1093/jnci/djj052

[B109] van't Veer LJ Dai H Vijver MJ van de He YD Hart AA Mao M Peterse HL Kooy K van der Marton MJ Witteveen AT Schreiber GJ Kerkhoven RM Roberts C Linsley PS Bernards R Friend SH Gene expression profiling predicts clinical outcome of breast cancer Nature 2002 415 530 536 10.1038/415530a 11823860

[B110] Wang Y Klijn JG Zhang Y Sieuwerts AM Look MP Yang F Talantov D Timmermans M Meijer-van Gelder ME Yu J Jatkoe T Berns EM Atkins D Foekens JA Gene-expression profiles to predict distant metastasis of lymph-node-negative primary breast cancer Lancet 2005 365 671 679 15721472 10.1016/S0140-6736(05)17947-1

[B111] Ghayad SE Vendrell JA Bieche I Spyratos F Dumontet C Treilleux I Lidereau R Cohen PA Identification of TACC1, NOV, and PTTG1 as new candidate genes associated with endocrine therapy resistance in breast cancer J Mol Endocrinol 2009 42 87 103 10.1677/JME-08-0076 18984771

[B112] Vendrell JA Bieche I Desmetz C Badia E Tozlu S Nguyen C Nicolas JC Lidereau R Cohen PA Molecular changes associated with the agonist activity of hydroxy-tamoxifen and the hyper-response to estradiol in hydroxy-tamoxifen-resistant breast cancer cell lines Endocr Relat Cancer 2005 12 75 92 10.1677/erc.1.00899 15788640

[B113] Vendrell JA Ghayad S Ben-Larbi S Dumontet C Mechti N Cohen PA A20/TNFAIP3, a new estrogen-regulated gene that confers tamoxifen resistance in breast cancer cells Oncogene 2007 26 4656 4667 10.1038/sj.onc.1210269 17297453

[B114] Musgrove EA Sergio CM Loi S Inman CK Anderson LR Alles MC Pinese M Caldon CE Schutte J Gardiner-Garden M Ormandy CJ McArthur G Butt AJ Sutherland RL Identification of functional networks of estrogen- and c-Myc-responsive genes and their relationship to response to tamoxifen therapy in breast cancer PLoS ONE 2008 3 e2987 10.1371/journal.pone.0002987 18714337 PMC2496892

[B115] O-charoenrat P Rusch V Talbot SG Sarkaria I Viale A Socci N Ngai I Rao P Singh B Casein kinase II alpha subunit and C1-inhibitor are independent predictors of outcome in patients with squamous cell carcinoma of the lung Clin Cancer Res 2004 10 5792 5803 10.1158/1078-0432.CCR-03-0317 15355908

[B116] Shridhar V Lee J Pandita A Iturria S Avula R Staub J Morrissey M Calhoun E Sen A Kalli K Keeney G Roche P Cliby W Lu K Schmandt R Mills GB Bast RC Jr James CD Couch FJ Hartmann LC Lillie J Smith DI Genetic analysis of early- versus late-stage ovarian tumors Cancer Res 2001 61 5895 5904 11479231

[B117] Wang Y Xu SR Lin FR Guo XN Ren JH Expressions of cyclin E2 and survivin in acute leukemia and their correlation Zhongguo Shi Yan Xue Ye Xue Za Zhi 2006 14 337 342 16638210

[B118] Muller-Tidow C Metzger R Kugler K Diederichs S Idos G Thomas M Dockhorn-Dworniczak B Schneider PM Koeffler HP Berdel WE Serve H Cyclin E is the only cyclin-dependent kinase 2-associated cyclin that predicts metastasis and survival in early stage non-small cell lung cancer Cancer Res 2001 61 647 653 11212263

[B119] Steg A Wang W Blanquicett C Grunda JM Eltoum IA Wang K Buchsbaum DJ Vickers SM Russo S Diasio RB Frost AR LoBuglio AF Grizzle WE Johnson MR Multiple gene expression analyses in paraffin-embedded tissues by TaqMan low-density array: application to hedgehog and Wnt pathway analysis in ovarian endometrioid adenocarcinoma J Mol Diagn 2006 8 76 83 10.2353/jmoldx.2006.040402 16436637 PMC1867577

[B120] Larson RA Le Beau MM Therapy-related myeloid leukaemia: A model for leukemogenesis in humans Chem-Biol Interact 2005 153-154 187 195 10.1016/j.cbi.2005.03.023 15935816

[B121] Cho NH Hong KP Hong SH Kang S Chung KY Cho SH MMP expression profiling in recurred stage IB lung cancer Oncogene 2003 23 845 851 10.1038/sj.onc.1207140 14647437

[B122] Ihmels J Collins SR Schuldiner M Krogan NJ Weissman JS Backup without redundancy: genetic interactions reveal the cost of duplicate gene loss Mol Syst Biol 2007 3 86 10.1038/msb4100127 17389874 PMC1847942

[B123] Nishiyama M Oshikawa K Tsukada Y-i Nakagawa T Iemura S-i Natsume T Fan Y Kikuchi A Skoultchi AI Nakayama KI CHD8 suppresses p53-mediated apoptosis through histone H1 recruitment during early embryogenesis Nat Cell Biol 2009 11 172 182 10.1038/ncb1831 19151705 PMC3132516

[B124] Kenny FS Hui R Musgrove EA Gee JMW Blamey RW Nicholson RI Sutherland RL Robertson JFR Overexpression of cyclin D1 messenger RNA predicts for poor prognosis in estrogen receptor-positive breast cancer Clin Cancer Res 1999 5 2069 2076 10473088

[B125] Satyanarayana A Kaldis P Mammalian cell-cycle regulation: several Cdks, numerous cyclins and diverse compensatory mechanisms Oncogene 2009 28 2925 2939 10.1038/onc.2009.170 19561645

[B126] Carthon BC Neumann CA Das M Pawlyk B Li T Geng Y Sicinski P Genetic Replacement of Cyclin D1 Function in Mouse Development by Cyclin D2 Mol Cell Biol 2005 25 1081 1088 10.1128/MCB.25.3.1081-1088.2005 15657434 PMC544006

[B127] Pearson W Rapid and sensitive sequence comparison with FASTP and FASTA Methods Enzymol 1990 183 63 98 full_text 2156132 10.1016/0076-6879(90)83007-v

[B128] Thompson J Higgins D Gibson T CLUSTAL W: improving the sensitivity of progressive multiple sequence alignment through sequence weighting, position-specific gap penalties and weight matrix choice Nucleic Acids Res 1994 22 4673 4680 10.1093/nar/22.22.4673 7984417 PMC308517

[B129] Felsenstein J PHYLIP -- Phylogeny Inference Package (Version 3.2) Cladistics 1989 5 164 166

[B130] Page R TREEVIEW: An application to display phylogenetic trees on personal computers Comput Appl Biosci 1996 12 357 358 8902363 10.1093/bioinformatics/12.4.357

